# Supplementary material for: Are carbon emissions trading and green financial instruments synergistic? -Comprehensive quantitative research based on content analysis
Source: PLoS One. 2024 Mar 7;19(3):e0298601. doi: 10.1371/journal.pone.0298601 (PMC10919601; doi:10.1371/journal.pone.0298601)
Supplement: S1 Data — (ZIP) [file pone.0298601.s001.zip › Data/地级市Z_F工作报告.zip!/╣π╢1⁄2/╔╟═╖╩╨/└┤╫╘╣1⁄2╓┌║┼í╛┬φ┐╦╩2╛▌═°í┐_2008.docx]

各位代表：

我代表市人民政府向大会作政府工作报告，请予审议，并请政协各位委员和其他列席人员提出意见。

一、2007年工作回顾

2007年，在省委、省政府和市委的正确领导下，市政府坚持以邓小平理论和“三个代表”重要思想为指导，认真贯彻党的十七大、省第十次党代会、粤东工作会议和市第九次党代会精神，全面落实科学发展观，振奋精神，真抓实干，全市经济蓬勃发展，社会繁荣稳定，开始转入科学发展、全面进步的新阶段，市十二届人大一次会议确定的经济社会发展主要目标顺利完成。

———经济全面发展

实现地区生产总值850.15亿元，增长13%，增速为1999年以来最快。地方财政一般预算收入42.49亿元，增长21.7%。

工业加快发展。新增规模以上工业企业360家，规模以上工业总产值首次突破千亿元大关，达1069.26亿元，增长16.8%，占全市工业总产值的61.3%，比上年提高1.2个百分点。全年电力供售量首次突破100亿千瓦时。自主创新能力增强。高新技术产品产值277亿元，增长21%。组建国家火炬计划龙湖输配电设备产业基地。新增中国名牌产品2个、驰名商标1件，国家出口免验产品2个、免检产品4个，新增专利授权3065项。韩国SK集团聚苯树脂等一批工业重点项目顺利投产。节能减排扎实推进，单位GDP能耗为全省平均水平的85.7%，位列全省第4低；二氧化硫减少排放量3920吨，超额完成省下达的减排任务。建筑业完成施工产值146亿元，增长18.3%，获“鲁班奖”2项。

农业稳步发展。完成农业总产值90.12亿元，增长3.5%。农业结构和区域化生产布局进一步优化，农业产业化步伐加快，现代效益农业框架初步形成。改造整治中低产田3.63万亩，农田基本建设力度为近年最大。农作物和禽畜良种率达到95%以上，农业标准化建设居全省前列。扩大鲍鱼、南美白对虾等特色品种生产规模，效益渔业建设不断推进。出台加快老区山区发展决定，落实项目48个，扶持老区山区发展力度加大。南澳县建成全省首个海上渔业生产救助通信网络。海门中心渔港前期建设和云澳中心渔港、达濠一级渔港申报工作积极推进。“万村千乡”市场工程、农村信息化示范村建设扎实推进。

服务业明显提升。港口物流业快速增长，实现货物吞吐量2301万吨、集装箱吞吐量59.39万标箱，分别增长14.2%和34%，再创历史新高。汕头机场旅客吞吐量111.5万人次，增长13.1%。旅游总收入61.44亿元，接待过夜游客556.85万人次，分别增长11.5%和11%。传统商贸业旺盛，社会消费品零售总额473.21亿元，增长18.1%。金融保险、证券、会展业加快发展。

———内外源型经济协调发展

招商引资成效显著。成功举办“4·25”春季招商引资经贸活动、粤台经济技术贸易交流会，赴美国、马来西亚等国家推介投资环境，一批项目签约、开工、投产。实际吸收外商直接投资1.72亿美元，增长22.9%；外贸出口总额39.12亿美元，增长12.3%。

民营经济活跃。个体工商户、私营企业总数达10.27万户，增长39.4%。实现民营经济增加值509.28亿元，增长12.8%，占GDP的59.9%。民营投资额占全市投资额61%。东方锆业、南洋电缆成功上市，宜华地产借壳上市，企业上市融资步伐加快。行业协会、商会作用日益增强。

国有企业改革步伐加快。完成市属国有企业改革49户，安置职工1072人。采取打包削债回购债权等办法，化解企业债务约35亿元，为加快国企改革创造有利条件。

———基础设施和市政设施建设加快推进

三大经济带前期工作进展顺利。东部城市经济带韩江河口治理规划获省批准，新津河及外砂河河口治理开发工程可研报告已报省审查，区域建设用海总体规划通过国家海洋局初审，新津河口启动片区控制性详规编制完成。工业经济带规划建设稳步推进，与深圳共建产业园区正式签约。生态经济带规划抓紧进行。

重点基础设施建设步伐加快。全社会固定资产投资额206.96亿元，增长17.1%。华能海门电厂一期1、2号机组工程、500千伏潮南输变电工程不断推进，南澳华能风电场二期工程建成投产。珠池港区5个万吨级泊位通过国家验收，正式投产。广澳港区对外国籍船舶开放获国务院正式批复，1个5万吨级泊位建成试投产，30万吨级原油码头、10万吨级集装箱码头、西防波堤项目前期工作全面启动。汕头湾过海隧道工程开始规划论证，南澳大桥、引韩供水、过海电缆“三大跨海工程”前期工作基本完成。积极配合厦深铁路建设，汕普、汕揭高速公路加紧筹建。机场路改造、陈沙公路建设加快。15个城乡水利防灾减灾项目建设全面提速，韩江下游出海口闸重建改建工程扎实推进。

市政基础设施建设取得新突破。中山东路改造工程竣工通车，金砂路东延、庐山路、澄海路、金环西路等一批市政连接路段和中泰、金泰立交桥地面工程完成建设，海滨路中段综!合市政工程、海滨路西延工程加紧建设，汕樟北路、天山北路续建、迴澜桥重建等工程开工建设。金凤路桥等项目拆迁工作取得突破。17个创模达标硬件工程加快推进，龙湖沟整治工程基本完成。

———社会环境明显改善

城市管理长效机制初步建立。中心城区市、区两级城市管理行政执法、园林绿化、卫生监督等管理体制得到理顺，市、区管理事项划分及市直部门职责进一步明确，市政设施规划建设管理、集贸市场综合管理、“门前三包”等制度逐步健全。市容环境整治全面展开。上下联动，齐抓共管，抓住热点难点，突破薄弱环节，深入开展声势浩大的市容环境、交通秩序、“五小”行业等专项整治，脏乱差现象得到有效治理，市容市貌有效改观，通过国家卫生城市复检。

生态环境建设得到加强。市区空气质量优良率99%。中心城区新增绿地面积229亩，改造绿地面积468亩，通过省园林城市验收。完成造林补植2.06万亩，沿海防护林体系建设效益明显。汕头海岸湿地国际示范区首期项目、国家“碳汇林”和“中日友好纪念林”启动建设。积极推进生态创建工作，新增市级生态示范区7个。加大饮用水源污染整治力度，市区饮用水源地水质达标率100%。

社会治安综合治理有序推进。开展专项行动，打击涉枪犯罪、“两抢一盗”等违法犯罪行为力度加大。集中整治治安重点地区和突出问题，社会治安防控不断强化。平安汕头建设扎实开展，58个社区获省首批“六好”平安和谐社区称号。

安全生产形势稳定。全市未发生较大以上安全生产事故，实现省下达的控制指标。《汕头市消防条例》正式实施。

———市场监管成效显著

产品质量和食品安全工作取得阶段性成效。开展玩具产品、农产品、食品加工、生猪屠宰等专项整治，12个100%目标任务顺利完成，监管长效机制初步建立。

清理经济户口效果明显。全市企业信息资源共享平台投入使用，清理漏征漏管1.46万户，取缔无证无照经营4632户，补办营业执照2.76万户。推行税收代征协管。启用即开型有奖发票，加强饮食娱乐行业税收征管。

土地和房地产市场管理力度加大。提高供地门槛，提升城市土地开发利用效率，节约集约用地。清理各类闲置、空闲土地208宗共8100亩，查处违法用地117宗1478亩。推进工业用地公开出让和集体建设用地使用权流转，土地市场建设进一步规范。启用中心城区房地产市场管理信息系统，开展专项整治，房地产市场平稳健康发展。

———社会各项事业取得新进步

就业和社会保障工作扎实开展。新增城镇就业岗位6.11万个，实现下岗失业人员再就业1.54万人，转移农村劳动力4.97万人，组织职业技能培训4.77万人。城镇登记失业率3.1%。社保基金征缴和监督进一步加强，征收社保五项基金14.49亿元，增长32.2%。改革完善养老金计发办法。企业离退休人员基本养老金人均月增加118元，建国前参加工作退休人员人均月增加700元。城乡居民最低生活保障标准和补助水平不断提高，受益群众逾8.53万人，基本实现应保尽保。提高残疾军人等重点优抚对象抚恤和生活补助标准。被征地农民养老保险试点工作正式启动，社会保险、社会救助制度不断完善。

教育、卫生、体育等事业全面发展。实施农村免费义务教育，惠及学生75.72万人。落实农村困难家庭学生生活费补助13.55万人。小学适龄儿童入学率99.5%，初中毛入学率达到100%以上，普及高中阶段教育工作全面启动。新改扩建中小学校舍48万平方米，增加学位3.17万个。中小学校C、D级危房改造工作基本完成，中小学新课程实施工作顺利开展。推动市第二技工学校升级，支持粤东高级技工学校改扩建。新型农村合作医疗制度覆盖所有镇村，参合农民272.43万人，参合率87.6%，住院补偿4.42万人次。社区卫生服务网络建设和农村规范化卫生站建设扎实推进。中心城区疾病预防控制实行市、区分级管理，市疾控中心新楼投入使用，重大传染病防控水平不断提高。国家乒乓球汕头训练基地正式启用。我市运动员获得世界大赛冠军4项、全国冠军20项。年度人口计划指标全面完成，人口自然增长率5.22‰。殡改工作进一步加强，省下达的年度殡葬管理目标任务顺利完成。

群众关心的热点难点问题逐步解决。列入省计划的6项农村饮水安全工程项目加快推进，36个村居15万群众“饮水难”问题得到解决。完成71个行政村通水泥路121公里工程。维修中心城区公房10.97万平方米，受益群众2737户。完成农村贫困户危房改造1633户，修建低保户住房100套。“新兴片区”旧城改造项目正式启动，金樟立交桥、大华路尾、浮东老村等旧城改造项目竣工面积20万平方米。个人住房公积金贷款工作顺利开展。加强对猪肉、液化气等重要商品的价格监管。居民消费价格指数上涨3.8%，保持在相对合理水平。中心城区居民人均可支配收入11716元，农村居民人均纯收入4604元，分别增长7%和4.5%。

———民主法制建设和精神文明建设稳步推进

民主法制建设得到加强。完成行政执法职权核准界定。加强对政府重大决策执行情况和重点建设项目进展情况的监督检查。成立行政服务中心，首批30个单位207个事项、5个便民项目进驻。受理机关效能投诉210件，办结195件。安排74个单位通过“民声热线”节目现场解答群众提问，畅通舆论监督渠道。办结人大代表议案（建议）88件、政协提案147件。提请市人大常委会审议法规草案2件，颁布政府规章9件、规范性文件15件。坚持重大问题集体研究，重大行政决策实行法律审查制度，涉及人民群众切身利益的重大决策实行听证制度和公示制度。出台政府工作部门行政首长问责暂行规定，强化部门职责。严格依法行政，审结行政复议案7件。加强预算执行、重点投资项目、专项资金审计，积极稳妥开展经济责任审计。推进政务公开、厂务公开、村（居）务公开和民主管理工作，基层民主政治建设不断加强。落实信访工作责任制，社会矛盾预防和化解机制进一步完善。

精神文明建设巩固发展。倡导和谐互助新风尚，扶弱济困、乐善好施的“关微”精神深入人心。第四次蝉联“全国双拥模范城”，南澳县首获“全国双拥模范县”称号。省文明城市创建工作积极推进。“共建美好新家园”主题教育活动、“书香飘万家”读书活动和“文化、科技、卫生”三下乡活动深入开展。《同一首歌》大型文艺演出、“奋进汕头”大型烟花晚会成功举办。潮剧《东吴郡主》荣获国家舞台艺术精品工程“精品提名剧目”称号。“南海Ⅱ号”古沉船抢救性保护工作有序开展。木雕、瓶内画、潮州音乐、抽纱、嵌瓷入选省第二批非物质文化遗产名录。文化艺术学校易地重建。“村村通广播”工程顺利推进。妇女儿童、残疾人、老龄、慈善和社会福利事业加快发展，外事、侨务、统计、人防、气象、地震、档案、方志、民族、宗教等工作取得新成绩。

各位代表！过去一年，我市经济社会发展取得的成绩，是省委、省政府和市委正确领导的结果，是各级人大及其常委会和人民政协监督支持的结果，是全市人民同心同德、奋力拼搏的结果，也是社会各方面支持和帮助的结果。在此，我代表市人民政府，向全市人民，向驻汕人民解放军、武警官兵、人民警察和各民主党派、人民团体、社会各界人士表示崇高的敬意！向关心和支持汕头建设的港澳台同胞、海外侨胞和国际友人表示衷心的感谢！

在肯定成绩的同时，我们清醒地认识到，我市经济社会发展仍存在不少困难和问题：一是粗放型经济发展方式尚未根本改变，自主创新能力较弱，产业竞争力不强，产业结构优化升级的任务很重；二是土地紧缺，资源和环境约束趋紧，可持续发展压力大，节能减排的任务很重；三是社会发展滞后于经济发展，群众在看病、出行、上学、居住等方面还有不满意的地方，部分城乡居民生活比较困难，改善民生的任务很重；四是一些长期积累的问题没有得到根本解决，基层矛盾比较突出，维护社会稳定的任务很重；五是政府职能转变还不适应科学发展的要求，服务意识不强、办事效率偏低问题依然存在，建设效能政府的任务很重。我们必须高度重视，将认真解决。

二、2008年经济社会发展主要目标

2008年，是我市贯彻落实十七大精神，实现“十年大发展”第一阶段奋斗目标的重要一年。我市既面临有利的发展环境，也面临新的挑战。一方面，国内经济社会发展势头良好，省委、省政府明确提出“加快发展以汕头为中心的粤东城镇群”、“加快珠三角地区产业向东西两翼和山区转移”，我市经济社会加速发展，总体实力进一步增强。另一方面，国际贸易摩擦加剧，国家宏观调控政策趋紧，我市投资拉力不足、融资困难、出口增速放缓、发展成本上升。对此，我们要始终保持清醒的头脑，把握机遇，迎接挑战，开拓创新，奋发进取，进一步提高驾驭经济社会发展全局的能力，全力推进汕头的振兴和崛起。

今年政府工作的指导思想是：深入贯彻党的十七大、省委十届二次全会、市委九届四次全会精神，以科学发展观为统领，紧紧围绕发展与和谐两大主题，按照建设粤东城镇群中心的发展目标，坚定不移解放思想，不断扩大改革开放，坚持好字优先、好中求快、能快则快，积极推进自主创新，调整优化经济结构，提升经济发展质量，提高行政效能，改善投资环境，大力发展民营经济、港口经济、园区经济，壮大经济总量，加强节能减排、环境保护、城市管理，切实改善民生、维护稳定、促进和谐，推动经济社会又好又快发展。

今年全市经济和社会发展的主要预期目标是：生产总值增长14%，单位生产总值能耗下降2.3%，二氧化硫和化学需氧量减排率分别为1.6%和4%，地方财政一般预算收入增长18.5%，全社会固定资产投资额增长18%，外贸出口总额增长13%，社会消费品零售总额增长16%，居民消费价格指数控制在4%以内，城镇登记失业率控制在3.3%以下，城镇居民人均可支配收入增长7%，农村居民人均纯收入增长5%，人口自然增长率控制在8‰以下。

三、全力推动经济又好又快发展

（一）加快构建现代产业体系

推动工业优化升级。加强产业引导，加大资金扶持力度，培育引进战略性产业，加快发展装备制造业、优势传统产业、高新技术产业。落实扶持规模以上工业企业加快发展措施，做强做大工业企业。运用高新技术改造和提升传统产业。推进公共科技创新平台建设，促进行业技术创新。加快国家玩具质量监督检验中心建设。加强产学研结合，推进与高等院校和科研院所的科技合作。精心打造澄海玩具礼品、龙湖外砂潮式工艺毛织服装、濠江工艺饰品等产业集群升级示范区，支持潮南纺织服装、家居内衣等特色产业集群优化升级。大力推进特色产业基地和专业镇建设。发展品牌经济，打造拳头产品，提升产品知名度和市场占有率。力争通过国家知识产权试点城市验收。

推进三大经济带规划建设。继续做好东部城市经济带相关项目前期工作。争取新津河口启动片区投资项目尽快公开招标、开工建设，抓紧塔岗围片区规划和前期工作，形成两头推进的格局。加快工业经济带石化园区、产业转移园区、数码园区、台商投资区规划建设，力争在大项目引进上实现突破，带动广澳临海片区、潮阳经济开发试验区、潮南井都陇田片区开发建设。抓紧完成生态经济带规划编制，为早日启动南滨片区和北山湾片区开发建设做好准备。

提升工业园区发展水平。鼓励现有工业园区二次开发，实现集约节约发展。启动建设高新区西片区和南片区。积极申请设立出口加工区、保税物流园区等特殊监管区域。调整提升金平、龙湖省级民营科技园上新水平。

大力发展港口经济。大力发展能源、石油化工、船舶修造、电动汽车、物流等临港产业。强化汕头港作为粤东港口群主枢纽港地位，重点建设广澳港区。开工建设广澳港区5万吨级石化码头，争取完成5万吨级航道工程，推进西防波堤、二期10万吨级集装箱码头、30万吨级原油码头、疏港铁路项目前期工作，争取西防波堤列入交通部“十一五”后期调整规划。启动马山港区3.5万吨级煤码头改扩建工程。加快华能海门电厂1、2号机组和电网送出工程建设，抓紧做好3、4号机组前期准备工作。争取丰盛海门电厂60万千瓦机组项目尽快核准。

加快发展现代服务业。以生产性服务业为重点，推进工艺玩具、纺织服装等专业批发市场的规划建设。发展现代运输服务业，拓展物流配送服务。提升传统服务业，推进易初莲花、沃尔玛等大型超市建设。积极推进会展设施规划建设，办好玩具展、服装展、食博会等特色展会。提高商务服务能力，发展和规范专业中介服务业。加强企业信用建设，建立健全信用担保体系，优化金融服务环境。增强城市辐射集聚功能，发展总部经济。打造海滨休闲度假品牌，完善旅游规划，强化旅游资源整合，加快蓝水星方特乐园等一批旅游项目建设。加强信息服务产业建设，完成本地电话号码升位工作。进一步做大做强建筑业，促进房地产业健康发展。

（二）扎实推进社会主义新农村建设

发展现代效益农业。调整优化农业结构，壮大农业龙头企业，培育农民专业合作经济组织。大力抓好农业标准化工作，提高农产品质量安全水平。发展农产品加工业、畜牧养殖业、现代渔业、现代林业，提高农业产业化水平。大力发展农业园区经济，建设牛田洋农业科技园以及澄海区下岱美村、潮南区上南村蔬菜现代化生产示范区等现代农业园区。推进规模化蛋鸡生产，争取高科技养虾项目落户。积极推动农田水利基本建设、农业机械化建设，全面完成小型水库除险加固，加快城乡水利防灾减灾工程建设，力争动工重建莲阳桥闸。加紧建设海门中心渔港，做好云澳渔港、达濠渔港申报国家级渔港建设项目工作。力争国家批准建设台湾农产品集散中心。

改善农村生产生活条件。进一步落实支农惠农政策，增加农民收入。加强集体建设用地管理，稳步推进老村改造。培育一批新农村建设示范村。抓紧落实扶持老区山区项目，发展老区山区经济，解决农村“一保五难”问题。组织农村公路建设攻坚战，基本实现村村通水泥路。加快农村消费品和农资新型流通体系建设，巩固发展“万村千乡”市场工程成果，方便农民消费。

（三）加强城乡规划建设做好城乡规划编制。认真实施《城乡规划法》。开展新一轮汕头市总体规划修编。积极配合国家、省做好主体功能区规划。完善市域规划和县域总体规划编制。全面开展市区控制性详规编制。理顺城乡规划管理体制，加强城乡规划管理，加大违法违章建设查处力度。

加快城市基础设施建设。推进汕揭、汕普高速公路、厦深铁路汕头段建设，以及国道324线潮阳路段和省道235线司神公路等国、省道路面改造和安保工程实施。完成海滨路西段、机场路改造、陈沙公路建设和海滨路中段道路绿化、灯光、观海平台工程。启动建设南澳“三大跨海工程”。抓紧金凤路桥拆迁工作，加快建设进程。继续加快一批城市主干道、出入口道路拓宽改造和市政道路连接段建设，推进汕樟北路、黄山路水浸街改造，黄山路、焰峰路续建和迴澜桥重建，落实国、省道四、五类桥梁安全隐患整治，启动金砂东路、大学路改造。加快汕头湾过海隧道、惠揭高速、登洪高速和濠洲路前期工作。

（四）深化改革开放

推进国企改革。完善企业法人治理结构，规范国有企业改制和国有产权交易行为。运用市场机制，引入社会资金参与国企改革。通过处置企业划拨（出让）用地等多种方式，盘活国企存量资产。统筹运作、打包处理国企债务。继续实施国有流通企业“减债解困工程”试点工作。尽快对条件成熟的国企实施政策性破产。全面完成县级供电企业体制改革。支持港务集团、超声集团等优势企业发展壮大。

促进民营经济发展。引导民营企业建立现代企业制度。引导和支持民营经济进入公用事业、基础设施和金融服务等行业。加大扶持力度，着力帮助企业解决融资、用地、用工等困难和问题。鼓励、扶持企业上市融资，争取新增3至5家上市公司。构建面向中小企业的创新创业服务平台。发挥商会、行业协会在沟通政府与企业关系、协调企业利益、促进产业协调发展等方面的积极作用。

提升招商引资水平。加强跟踪服务，提高签约项目履约率。鼓励全民创业，积极争取海内外投资者投资兴业。精心策划各类招商引资经贸活动，重点推介东部城市经济带、工业经济带、产业园区以及一批基础设施、市政设施项目。办好首届粤东侨博会暨经贸洽谈会。

转变外贸增长方式。优化对外贸易结构，大力发展服务贸易和技术贸易，加快加工贸易升级转型。支持、帮助企业“走出去”拓展海外市场，扶持拥有自主知识产权、自主品牌和高附加值产品出口。做好水产品、玩具等商品应对国际贸易摩擦工作。充分利用原产地优惠政策扩大出口。扩大我市急需的能源、重要原材料和先进技术设备进口。推进台湾渔船自捕水产品贸易发展。表彰奖励进出口大户。加紧启动广澳港区对外开放一类口岸运作，规范进出口秩序，完善口岸通关环境。

加强区域合作。积极融入海峡西岸经济区，争取享受国家优惠政策，进一步加强对台经贸合作。巩固发展与港澳的合作关系，积极参与闽粤赣协作区、泛珠三角区域、中国———东盟自由贸易区经济合作，推动与友好城市的合作交流。深化粤东四市经济社会协作，争取在产业布局、基础设施建设、资源开发、旅游等方面合作有实质性进展。

（五）加强资源能源节约和生态环境保护

强化节能减排。落实节能减排目标责任制、考核制和问责制，强化执法监察。积极引导发展高科技、高附加值、低能耗、少污染行业，严格抑制高耗能行业，坚决淘汰落后生产能力，推进电力、造纸、印染等产业的节能降耗和污染减排，推广清洁生产。开展资源综合和循环利用，重点支持能源资源节约和综合利用新技术、新工艺、新设备的研究开发。抓好贵屿镇循环经济试点工作，加强莲花山废矿综合整治。

全力推进创模整改达标。完成龙珠水质净化厂技改和扩建及配套管网建设，开工建设北轴污水处理厂和雷打石生活垃圾焚烧发电厂，推进污水处理设施、垃圾处理场、市环境监控中心等硬件设施建设，加强城市河沟污染整治，强化工业污染源监管，力争通过创模整改达标验收。加大垃圾处理费征收力度。扎实推进滩涂红树林、水源涵养林等生态公益林建设，加强汕头湾生态环境保护，大力发展生态文明。

节约集约用地。继续推进新一轮土地利用总体规划修编，科学合理配置土地资源。深化土地管理制度改革，转变土地利用方式。加大盘活、处置闲置土地力度，增加土地储备，确保重点项目和产业建设用地。完善土地市场建设，规范土地交易。落实最严格的耕地保护制度，严肃查处违法违规用地行为。

（六）强化经济管理

加强财税管理。充分利用企业信息资源共享平台，大力清理经济户口和无证无照经营，推行税收代征协管，做到应收尽收。优化企业纳税服务。平稳实施新企业所得税法。建立政府资源有偿使用机制，进一步盘活政府资源。深化市、区财政体制改革，优化财政支出结构，加强支出绩效评价。

坚持安全发展。积极稳妥做好城信社退出市场工作，推进农信社改革试点，探索信托投资公司最终处置，防范化解金融风险。全面加强产品质量安全工作，强化对涉及人民健康和安全的食品、药品等重点产品的监管，构建保障产品质量安全的长效机制。加强市场价格调节和监管，完善价格调节基金制度，推进环境资源价格改革，做好粮食和副食品等重要商品的市场供应和调控，加强煤电油运保障工作，保持价格总水平基本稳定。严格落实各级政府安全生产监管责任和企业安全生产工作责任，深入开展危险化学品、交通、消防、建筑施工等重点行业和领域的安全隐患排查治理，依法严厉查处各类安全生产违法违规行为，有效遏制重特大事故发生。

四、积极促进社会和谐

（一）大力发展社会事业

优先发展教育。全面实施城乡免费义务教育，巩固提高普及九年义务教育水平。加大力度发展高中阶段教育，加快中等职业技术教育发展步伐，鼓励社会力量积极参与举办普通高中和中等职业技术学校，大幅度提高高中阶段教育毛入学率。加快校舍建设步伐，进一步改善农村学校办学条件，推进学校布局结构调整。规范民办教育，重视学前教育。完善助学机制，落实贫困家庭学生资助政策。改善教师待遇，加强教师培训，提高师资队伍整体素质。争取汕头职业技术学院通过国家评估，支持粤东高级技工学校改扩建工作，支持汕头大学提高办学水平。

加强人才队伍建设。重视现有人才的培养和使用，尊重人才、关心人才、留住人才。出台人才引进政策措施，办好在职人才的专业教育和技能培训，拓宽人才资源开发与交流合作渠道，健全激励和考核机制，建立完善科学的人才评价制度。

完善公共医疗卫生体系建设。全面实施和巩固完善新型农村合作医疗制度，实现农村常住人口基本享有合作医疗制度保障。抓好规范化村卫生站、镇卫生院和社区卫生服务体系建设，方便群众就医就诊。加大力度打击各种非法行医行为，规范医疗市场秩序。加强疾病防控，提高全民健康水平。

加强和谐文化和精神文明建设。全面开展创建省文明城市活动。深入开展现代公民教育，加强未成年人思想道德建设。弘扬“关微”精神，营造互助互爱的社会氛围。征集“汕头形象”歌曲，展示城市精神风貌。完善公共文化服务体系，大力发展文化事业和文化产业，力争在文化创意业、动漫业、文化旅游业上有新突破。推进潮剧改革和发展，办好国际潮剧节。筹建市美术馆，解决市文化宫建设等一批遗留问题，启动市档案馆新馆规划筹建工作。重视文物和非物质文化遗产。继续做好“南海Ⅱ号”古沉船保护和潮汕侨批申遗工作。抓紧隆都镇前美村申报中国历史文化名村工作。广泛开展全民健身活动，增强市民体质。推进体育中心、体育公园规划建设。积极配合奥运会火炬传递活动。做好双拥工作，促进军政军民团结。

（二）注重改善民生

扩大就业再就业。贯彻实施《劳动合同法》，促进企业依法用工，维护劳动者合法权益。继续实施积极就业政策，推进创业富民工程，创建“充分就业社区”，组织实施“三年30万”就业援助计划等专项就业服务，着力帮助城乡就业困难人员就业。新增就业岗位6万个。

完善社会保障制度。扩大社会保险覆盖面，启动做实养老保险个人账户和视同缴费年限确认建账工作，提高企业离退休人员养老待遇。加强社保基金监督管理。全面推进城镇职工基本医疗保险，开展城镇居民基本医疗保险试点工作。提高企业职工最低工资标准。健全社会救助体系，提高城乡低保补助水平，确保应保尽保。做好优抚安置工作，大力发展社会福利和慈善事业。

解决群众切身利益问题。加快发展公共交通，增加公交线路和车辆投放，扩大公交覆盖面，方便群众出行。突出抓好停车场建设，缓解停车困难。加大财政投入，加快解决城镇低收入家庭住房困难，加紧建设廉租住房和经济适用房，推进住房货币化分配。加快潮阳、潮南引韩供水工程建设。

（三）提高社会管理水平

加强城市管理。推进城市管理重心下移，依法委托或授权给区、街道更多的管理职权，形成以市级为主导，区、街道、社区居委会为主力，群众积极参与的城市管理新格局，着力构建城市管理长效机制，巩固发展国家卫生城市成果。坚持不懈地加强城市环境综合整治，防止回潮和反复。加大资金投入，加快环卫设施升级和扩容。加强出租车、人力三轮车管理，整治交通运输秩序。规范户外广告、夜景灯光管理。增加绿量，提高绿化管养水平，为2009年创建国家园林城市打下基础。

切实维护社会稳定。加强社会治安综合治理，加快群防群治工作和治安防控体系建设，依法严厉打击“两抢一盗”等各类刑事犯罪活动，打击各类破坏公共设施和败坏社会风气的行为，确保社会安定有序。抓紧建设社会治安视频监控系统，提高治安动态监控能力。加强对网吧、校园周边环境的整治，大力做好预防青少年违法犯罪工作。高度关注群众信访问题和群体性事件，拓宽与人民群众的联系渠道，探索建立信访、复议与维稳的新机制，及时解决群众反映强烈的问题，妥善处理人民内部矛盾。充分发挥仲裁、调解等法律机制在化解社会矛盾，维护社会稳定中的作用。完善应急管理机制，有效预防和处置各种突发事件。

大力推进社区建设。理顺关系，完善公共服务设施，构建多元互动的城市社区组织管理体系，打造“六好”平安和谐社区。认真做好村（居）委换届选举，推进村（居）务公开和民主管理工作，维护基层和谐稳定。加强社会组织的建设和监督管理。

加强人口综合管理和服务。稳定低生育水平，积极开展生育关怀行动，提高出生人口素质。建立健全层级动态管理机制，依法征收社会抚养费，兑现计生奖励政策。全力推进濠江、潮阳、潮南转化上升为计划生育二类地区。加强流动人口计划生育管理和服务。完成殡葬管理任务目标。

五、切实加强政府自身建设

进一步解放思想。从时代发展的要求和汕头的实际出发，进一步增强忧患意识，坚持解放思想，弘扬特区精神，树立世界眼光，创新观念，创新思路，创新方式，以新一轮思想大解放推动新一轮大发展。落实行政首长问责制，依法管理经济社会事务，解决社会矛盾，提高政府的执行力和公信力。大兴调查研究之风，实事求是、求真务实。精简文件，精减会议，切实改进文风会风。加强公务员教育和管理，开展创建好班子活动，打造一支政治坚定、业务精通、清正廉洁、作风优良的公务员队伍，努力争当实践科学发展观的排头兵。

全面推进依法行政。提高立法质量，重点加强促进社会发展、加强社会管理、规范政府行为等方面的立法，推进“法治政府”建设步伐。全面落实行政执法责任制，规范行政执法，强化行政执法监督，抓好行政复议。自觉接受人大及其常委会的监督和政协的民主监督，认真执行人大决议，办理好人大代表议案、建议和政协委员提案。发挥政府公报、政府网站、政府新闻发布会等功能，重视新闻舆论和社会公众监督，支持监察、审计部门依法独立监督，提高行政透明度。

改善政府管理方式。建立健全重大决策的规则、程序和重大决策法律审查制度，实行依法决策、科学决策和民主决策。完善政府机构设置，理顺政府部门职责，合理划分市、区（县）的事权和财权，切实解决职能交叉、职责不明确、权责脱节问题。推进第五批行政审批制度改革，减少政府对微观经济运行的干预。完善行政服务中心建设，尽快完成第二、三批行政审批事项进驻工作，进一步提高办事效率。

强化公共服务职能。适应社会主义市场经济发展的新形势，努力创造公平竞争的市场环境、稳定透明的政策环境、规范健全的法制环境、优质高效的服务环境。更加注重发挥市场优化资源配置作用，更加注重依靠服务和政策促进经济发展。加强公共服务，把经济管理的主要精力更多地放到为市场主体服务上来，把财力物力更多地集中到公共服务上来，把政策的着力点更多地放到解决民生问题上来，为企业排忧解难，为基层多办实事，为群众多谋利益。

切实加强廉政建设。严格执行党风廉政建设责任制和领导干部廉洁从政的各项规定。加强对行政审批、财政资金分配、专项资金管理、工程招投标等重点领域的监管，坚决纠正损害群众利益的不正之风，重点解决生态环境保护、食品药品质量、安全生产、征地拆迁以及教育、医疗等方面群众反映强烈的问题。深入开展专项治理，坚决查处违法违纪案件。

各位代表，全面完成全年目标任务，实现对人民的庄严承诺，我们使命光荣，责任重大。让我们高举中国特色社会主义伟大旗帜，紧密团结在以胡锦涛同志为总书记的党中央周围，以科学发展观统领经济社会发展全局，在市委的正确领导下，紧紧依靠全市人民，励精图治，真抓实干，为加快实现汕头的全面振兴和崛起而努力奋斗！
